# Metagenomic study reveals hidden relationships among fungal diversity, variation of plant disease, and genetic distance in *Cornus florida* (Cornaceae)

**DOI:** 10.3389/fpls.2023.1282188

**Published:** 2024-01-11

**Authors:** Andrew Pais, Jean Ristaino, Ross Whetten, Qiu-Yun (Jenny) Xiang

**Affiliations:** ^1^ Department of Plant and Microbial Biology, North Carolina State University (NCSU), Raleigh, NC, United States; ^2^ Department of Entomology & Plant Pathology, North Carolina State University, Raleigh, NC, United States; ^3^ Emerging Plant Disease and Global Food Security Cluster, North Carolina State University, Raleigh, NC, United States; ^4^ Department of Forestry and Environmental Resources, North Carolina State University (NCSU), Raleigh, NC, United States

**Keywords:** metagenomics, GBS (genotyping-by-sequencing), *Cornus florida* (flowering dogwood), pattern of foliar fungal diversity, genetic differentiation

## Abstract

**Introduction:**

Understanding patterns of plant-microbe interactions across plant species and populations is a critical yet poorly characterized aspect in the field of plant pathology. Microbial DNA sequences present as contaminants in omics data of plants obtained using next-generation sequencing methods provide a valuable source to explore the relationships among endophytic microbial diversity, disease and genetic differentiation of host plants, and environmental variation, but few such studies have been conducted. The flowering dogwood tree (*Cornus florida* L.), an ecologically important species in North America, is threatened by powdery mildew and dogwood anthracnose diseases, and knowledge of the microbial diversity harbored within genetically and environmental distinct populations of this species remains largely unknown.

**Methods:**

We conducted a metagenomics study utilizing the sequences of RAD-tag/genotype-by-sequence libraries from leaf tissues of *C. florida* to examine such host-fungus interactions across the dogwood's US range. We performed various combinations of alignments to both host and pathogen genomes to obtain filtered sets sequences for metagenomics analysis. Taxonomic assignments were determined on each filtered set of sequences, followed by estimation of microbial diversity and correlation to environment and host-genetic variation.

**Results:**

Our data showed that microbial community composition significantly differed between visually healthy and diseased sites. Several microbial taxa known to interact with dogwood were identified from these sequences. We found no correlation between microbial diversity and relative abundances of sequences aligning to draft genomes of either pathogen causing powdery mildew or dogwood anthracnose. We found a significant relationship between differences of fungal communities and geographic distances of plant populations, suggesting roles of environments in shaping fungal communities in leaf tissues. Significant correlations between the genetic differentiation of plant samples and fungal community dissimilarity (beta diversity) were also observed in certain sets of our analyses—suggesting the possibility of a relationship between microbial community composition and plant genetic distance. This relationship persisted in significance even after controlling for significant effects of geographic-bioclimatic variation of microbial diversity.

**Discussion:**

Our results suggest that both genetics and the environment play a significant role in shaping foliar fungal communities. Our findings underscore the power of leveraging hidden microbial sequences within datasets originally collected for plant genetic studies to understand plant-pathogen interactions.

## Introduction

Mining endophyte community data from omics data in plant hosts is providing new opportunities to understand the environmental and microbial dynamics that may influence plant health and genetic differentiation. If properly analyzed, latent signatures of microbial composition within existing large molecular datasets can reveal important information for promoting plant growth and controlling plant disease and pests (Kaul et al., 2016). Metagenomic reanalysis of plant-associated sequences (LaBonte et al., 2018; Han et al., 2021^1^) can inform how patterns of microbial co-occurrence within plant hosts, help to track disease pathogens, and predict ecological roles of endophytes, which may be applied as novel biological control products for the management of plant pathogens (Akram et al., 2023). Even plant expression datasets of host plant species may reveal interesting signatures of endophyte community composition (Chen et al., 2022) while simultaneously revealing how endophytes can limit infection by plant pathogens through induced expression of systematic defenses by the plant host (Oukala et al., 2021; Collinge et al., 2022). While such studies explore how endophyte diversity impacts plant responses to disease and environment, the question of how plant host genetics is related to the microbial diversity within natural plant populations remains unanswered in many study systems due to the difficulty in controlling for the effects of geographic and bioclimatic variation. The advances and questions posed were applied to this study of the woody plant species known as the flowering dogwood (*Cornus florida* L.), which faces numerous ecological challenges in its native habitat.

*C. florida* (the flowering dogwood tree) is an ecologically important species in part due to calcium it provides for eastern United States forests (Jenkins et al., 2007; Borer et al., 2013), and it also yields up to 30 million dollars in annual sales in horticultural markets (NASS, USDA Census of Agriculture 2007). However, the species is increasingly threatened by epidemics caused by fungal plant pathogens, which can also concomitantly impact pollinators and fruit dispersers of flowering dogwoods (Rossell et al., 2001). Powdery mildew, caused historically by *Phyllactinia guttata* (Wallr.) Lév. and more recently by *Erysiphe pulchra* (Cook & Peck, Braun and Takamatsu 2000), and dogwood anthracnose, caused by *Discula destructiva* (Redlin, 1991), have negatively impacted the growth and reproduction of natural populations of *C. florida* (Daughtrey et al., 1996; Klein et al., 1998) and have led to diminishment of natural populations. Moreover, cultivated varieties have become more expensive to maintain as the need for fungicidal treatments has driven costs of crop management up 15-fold since the 1970s (Li et al., 2009). The life histories of each fungal pathogen and its preferred environment most likely contribute to different but overlapping distributions between dogwood anthracnose and powdery mildew in the eastern US.

Since its introduction from Asia to eastern North America approximately four decades ago (Pirone, 1980; Miller et al., 2016), dogwood anthracnose has severely decimated flowering dogwood populations (Hiers and Evans, 1997; Williams and Moriarity, 1999; McEwan et al., 2000; Jenkins and White, 2002), inflicting up to 94% mortality rates in certain areas (Sherald et al., 1996). However, it is most severe in northern and mountainous regions (Oswalt et al., 2012) where cooler and wet conditions promote the proliferation of *D. destructiva* (Chellemi and Britton, 1992; Daughtrey et al., 1996; Hiers and Evans, 1997).

Natural populations of dogwood may still be challenged by other epidemics caused by powdery mildew fungi. Leaves with powdery mildew infection are more prone to desiccation and leaf scorch (Windham et al., 1998) and can suffer from stunted growth, lower reproductive output, and in some cases death (Hartman, 1998; Daughtrey and Hagan, 2001). Powdery mildew caused by *E. pulchra* was first observed in 1994 in the southeast US (Hagan and Mullen, 1997) and may be another example of an exotic pathogen transferred from an Asian sister species of *C. florida* (Takamatsu et al., 1999; Bai et al., 2014). Despite extensive documentation of *E. pulchra* in plant nurseries (Mmbaga, 2000; Li et al., 2009) and some natural populations in Alabama, Tennessee, and Connecticut (Hagan and Mullen, 1997; Klein et al., 1998; Smith, 1999; Williamson and Blake, 1999), the hypothesis that powdery mildew occupies a widespread distribution in the US has not been fully tested for natural populations on the periphery of *C. florida*’s range such as populations in the Midwest.

The extent to which the last epidemic of powdery mildew has spread into previously anthracnose-free areas (or into areas already plagued by anthracnose) is unknown—necessitating continual tracking of range expansion for *E. pulchra* and *D. destructiva*. While the disease phenotype for powdery mildew becomes readily apparent during the summer, infected buds and leaves are difficult to identify visually for the first few months after inoculation in early spring. The disease cannot be visually observed without magnification until conidiophore growth becomes dominant, usually manifesting itself by summer as a white powdery covering and shriveled leaves. Moreover, the diagnostic presence of chasmothecia is not readily observed until fall. Spore capture methods (Mmbaga, 2002), fungal culture, and single-gene amplification procedures (Mmbaga et al., 2004; Zhang et al., 2011; Miller et al., 2016), currently used to monitor the occurrence of powdery mildew and dogwood anthracnose, are costly and time prohibitive for tracking diseases along the entire distribution of *C. florida*. In addition, dogwood anthracnose symptoms (i.e. leaf blotting readily apparent year round upon first bud break) can be easily confused with other cosmetic diseases such as spot anthracnose caused by *Elsinoe cornii* (Jenkins and Bitancourt, 1948) and *Septoria cornicola* (Farr, 1991). Given the limitations for monitoring the occurrence of pathogens such as *E. pulchra* and *D. destructiva*, here we pursued innovative metagenomics tools to indirectly assess the occurrence of fungal pathogens and other phyllosphere fungi *in vivo* through analyses of the microbial sequences in the GBS data of *C. florida* from our previous two studies (Pais et al., 2017; Pais et al., 2020). Microbial sequences contained in omics data of plants are the contaminant sequences of plants and often removed by researchers without further usage. We used these sequences for an innovative metagenomic study (Handelsman et al., 1998; Chen and Pachter, 2005) to further explore the relationships among fungal diversity, plant disease, plant population genetic distance, and the geographic distance/environment.

## Materials and methods

### Sampling and metadata at collection sites

In two previous studies, we applied the genotype-by-sequence (GBS) method (Peterson et al., 2012) to evaluate the genetic diversity of *C. florida* populations (Pais et al., 2017; Pais et al., 2020). In this work, we applied those paired-end (PE) reads from our previous Illumina-sequenced GBS datasets and aligned them exclusively to the genome of *E. pulchra* or *D. destructiva* (and checked that they did not also align to the draft genome of *C. florida*) to pull out microbial sequence reads *in silico*. With this metagenomics approach (Handelsman et al., 1998; Chen and Pachter, 2005) and these “fungal” sequences, we investigated the relationship between environmental factors, powdery mildew and dogwood anthracnose occurrence, microbial diversity, and taxonomic assignments made on flowering dogwoods sampled over the eastern range of *C. florida* in the US.

The sampling of trees and extraction of DNA from leaf samples were described in Pais et al. (2017) and Pais et al. (2020) and included 312 samples that had successful alignments to the available draft genomes of two important fungal pathogens associated with *C. florida* (described further in *Sequencing libraries and alignment to E. pulchra and D. destructiva*). These 312 samples served as the basis for two different approaches of metagenomics study being compared as described further in subsequent sections. As a summary of sampling from Pais et al. (2017), trees (180) of *C. florida* were sampled from the North Carolina Mountain, Piedmont, and Coastal Plain regions (**Table 1**). Mountain populations included two to four sites from the Smoky Mountains National Park and Pisgah National Forest (NC-SM1, NC-SM2, NC-PI1, and NC-PI2). Duke Forest and Umstead State Park (NC-DK and NC-UM) represented the Piedmont, and the Coastal Plains was represented by populations from Nags Head Woods and Croatan National Forest (NC-NW and NC-CF). For samples collected in Pais et al. (2017), approximately 15-31 samples were collected per site, and sites in close proximity were considered part of a larger population. Each of the three regions was represented by two populations with similar ecological niches. Healthy leaves were collected in the early to mid-summer of 2012. Samples were placed on ice during the collecting trip and stored in a -20°C freezer in the lab until DNA extraction. DNA was extracted in the subsequent fall season (see details below in *Sequencing libraries and alignment to E. pulchra and D. destructiva*).

**Table 1:** Summary of location, sampling, and conditions of each collection site sampled for *Cornus florida* leaf tissue.

Site^a^	Latitude	Longitude	Date Collected	Trees sampled	Anthracnose disease visually observed at site	Confirmation of dogwood anthracnose disease in county	Mean temp. at month collected (°C)	Precipitation total at month of collection (mm)
NC-PI1	35.25	-82.74	May 2012	PI(1-15); n=15	Yes	Yes	18.34	110.31
NC-SM1	35.56	-83.32	May 2012	SM(1-15); n=15	Yes	Yes	16.92	120.84
NC-SM2	35.24	-83.24	May 2012	SM(16-30); n=15	Yes	Yes	16.80	147.69
NC-CF	34.93	-77.14	June 2012	CF(1-30)+CF(2) replicate; n=31	No	No	23.17	67.92
NC-DK	36.00	-78.97	June 2012	DK(1-23,25-30)+DK(22) replicate; n=30	No	No	22.75	89.05
NC-PI2	35.49	-82.63	June 2012	PI(16-30); n=15	Yes	Yes	19.81	37.00
NC-UM	35.85	-78.76	June 2012	UM(1-5,7-30); n=29	No	No	23.27	84.84
NC-NW	35.98	-75.66	July 2012	TNC(1-30); n=30	No	Yes	27.37	251.13
FL-SO	29.67	-81.96	May 2015	SO-COFL(3-17); n=15	No	No	24.44	99.24
SC-PE	33.83	-81.20	May 2015	SC-PE(1-10); n=10	No	No	22.10	34.69
NC-RW	34.49	-79.20	June 2015	NC-RW(1-15); n=15	Yes	No	27.30	215.00
SC-BR	33.47	-79.50	June 2015	SC-BR; n=10	No	No	26.93	95.35
KS-GAL	37.04	-94.64	July 2015	KS-GAL(1-4,6-9,11-15); n=13	No	No	27.16	148.68
MI-BD	42.90	-85.36	July 2015	MI-BD(1-6,8-10,12-15); n=13	Yes	Yes	21.04	94.90
MI-FC	42.33	-85.33	July 2015	MI-FC(1-4,7-14); n=12	Yes	Yes	20.82	133.67
MO-CC	36.82	-94.30	July 2015	MO-CC(1-10); n=10	No	No	26.45	218.83
MO-LO	38.21	-92.62	July 2015	MO-LO(1-6,9-14); n=12	No	No	26.05	209.98
MO-PC	36.70	-93.61	July 2015	MO-PC(1-12,14-15); n=14	No	No	25.94	388.77
OH-AK	41.13	-81.57	July 2015	OH-AK(1-2,4-9); n=8	Yes	Yes	22.13	69.00

a Each collection site’s identifier is prefixed by its US state of origin.

**Figure 1:**
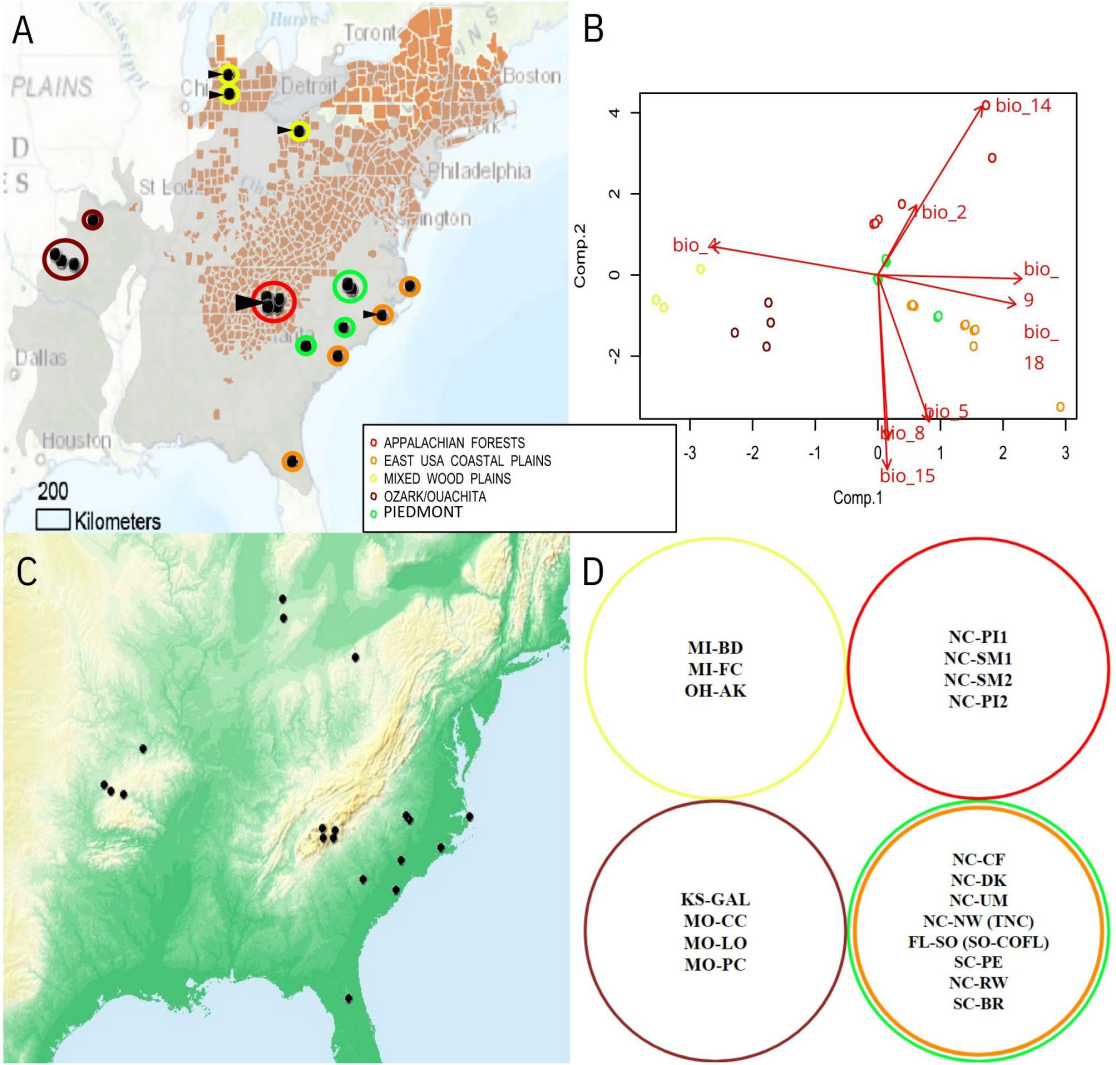
**(A)** Map of sampling locations across Flowering Dogwood (*Cornus florida*) range (gray), including: Ozark/Ouachita gallery forests (Missouri; maroon), Mixed Wood Plains (Michigan and Ohio; yellow), Appalachian mountains (North Carolina; red), Piedmont subpopulations (North Carolina and South Carolina; green), and eastern Coastal Plains (North Carolina, South Carolina, and Florida; orange). Counties highlighted in red have verified reports of dogwood anthracnose, according to US Forest Services whereas sites with visual observation of anthracnose disease (e.g. leaf blotting and necrosis) are indicated with triangular arrows. Climatic relationships of sampling sites depicted as **(B)** PCA of bioclimatic variables. Variable set reduced of collinearity prior to PCA is depicted as joint-biplot with line lengths proportional to degree of correlation to top two principal components. Bioclim variables (http://www.worldclim.org/) include: mean diurnal range of temperature (Bio2), temperature seasonality (Bio4), warmest month maximum temperature (Bio5), mean temperature of wettest quarter (Bio8), mean temperature of driest quarter (Bio9), driest month precipitation (Bio14), precipitation seasonality (Bio15), and precipitation of warmest quarter (Bio18). **(C)** Tinted hillshade terrain overlay to represent elevation. Darker green shades represent locations closer to sea level while darker orange-shaded regions are at higher elevations. **(D)** Collection sites’ membership to genetic clusters inferred from Pais et al., 2020 [colors correspond to same ecoregions as parts **(A, B)**].

Sampling in Pais et al., 2020 included samples from studies of Hadziabdic et al. (2010), Call et al. (2016), and Pais et al. (2017) as well as additional samples collected in the summer of 2015 from the eastern Coastal Plains and Carolina Piedmont, the Midwest, and west of the Mississippi River (n=132) (**Table 1**). In the southeast, we collected from central Florida (FL-SO) (n=15), two sites in South Carolina (SC-BR and SC-PE) (n=20), and the southeast portion of North Carolina (NC-RW) (n=15) (**Figures 1A, C**). In addition to the southeast United States, we also sampled from a portion of the Mixed Wood Plains surrounding the Great Lakes as well as the Ozarks and gallery forests west of the Mississippi river (**Figures 1A, C**). There were two sites sampled from Michigan (MI-BD and MI-FC) (n=13 and n=12, respectively) and one site from northern Ohio (OH-AK) (n=8). There were four sampling sites from west of the Mississippi in Missouri and southwest Kansas (MO-LO, MO-PC, MO-CC, and KS-GAL) (n=12, n=14, n=10 and n=13, respectively).

Differences in disease and environment among sampling sites were characterized with visual observations of dogwood anthracnose at the sampling site, records of precipitation and mean temperature at the month of collection, and bioclimatic data interpolated from GIS. We categorized sampling sites into groups that had visual evidence for the absence-presence of dogwood anthracnose following the convention of Hadziabdic et al. (2010) and Call et al. (2016). Dogwood anthracnose could be distinguished from other leaf blotting diseases by the red-purple borders of lesions on browning or necrotic leaves. Also, initial infection could often proceed from the leaf blade and petioles to stems and trunks—resulting in cankers, lower branch dieback, and often death within two to three years (Jenkins and White, 2002). In contrast, while powdery mildew might also contribute to leaf necrosis later in the growing season, the only noticeable symptom of this disease during our time of collection was a white powdery fungal mycelium on the surface of leaves, which was readily apparent at all collection sites. The samples collected by Hadziabdic et al. (2010) and Call et al. (2016) were not examined individually for disease given potential differences in sampling leaf tissue among collectors, but were instead designated as belonging to collection sites with or without visual evidence of disease.

Since seasonal conditions at time of collection might influence the ability to observe dogwood anthracnose disease, estimates of precipitation and mean temperature at month of sampling were extracted for each collection site using data available by the PRISM Climate Group (http://prism.oregonstate.edu). For the 19 bioclim variables describing climatic features derived from seasonal averages of precipitation and temperature from 1960-1990 [mean annual temperature (Bio1); mean diurnal range (Bio2); isothermality (Bio3); temperature seasonality (Bio4); max temperature for warmest month (Bio5); minimum temperature of coldest month (Bio6); temperature annual range (Bio7); temperature mean of wettest quarter (Bio8); temperature mean of driest quarter (Bio9); temperature mean of warmest quarter (Bio10); temperature mean of coldest quarter (Bio11); annual precipitation (Bio12); precipitation of wettest month (Bio13); precipitation of driest month (Bio14); precipitation seasonality (Bio15); precipitation of wettest quarter (Bio16); precipitation of driest quarter (Bio17); precipitation of warmest quarter (Bio18); and precipitation of coldest quarter (Bio19); Hijmans et al., 2005; http://www.worldclim.org/bioclim], high resolution data (30 arc-seconds or approximately 1 kilometer) were extracted via Arc-GIS based on all available sampling sites’ coordinates, which also included samples from Hadziabdic et al. (2010) and Call et al. (2016). Climatic data was extracted for these sites to help model *C. florida*’s adaptive landscape. The resulting dataset was parsed of multicollinearity by iteratively removing variables until remaining variables had variance inflation factor (VIF) scores less than ten, in accordance with standard procedure to remove correlated variables (Hair et al., 1995). We applied principal component analysis (PCA) on the remaining set of bioclim predictors to provide principal component scores characterizing the location of this study’s sampling sites in the context of the adaptive landscape (**Figure 1B**). In order to understand the influence of the environment on the distribution of dogwood disease and microbial diversity, the first two components’ scores (envPC1 and envPC2) in addition to precipitation and mean temperature at collection were applied to downstream analyses evaluating the relationships among microbial diversity, environmental conditions, and genetic differentiation of the flowering dogwood samples. Even though elevation and latitude were not directly modeled, their effects were captured by climate predictions used to formulate bioclim predictors and ultimately envPC scores.

While most downstream analyses required individual-level and collection site averages, collection sites were also categorized into genetic clusters of flowering dogwood populations inferred from Pais et al., 2020 for downstream comparisons in addition to categorizing collection sites into categories of healthy vs. diseased locations. The first genetic cluster consisted of populations sampled in the Ozarks and Ouachita gallery forests of North America (KS and MO states; **Table 1**; **Figures 1A, D**). The second designated cluster consisted of populations sampled primarily in the Mixed Wood Plains close to the Great Lakes region of North America (MI and OH states; **Table 1**; **Figures 1A, D**). The largest genetic cluster of *C. florida* samples was from the Piedmont and Coastal Plain regions of the southeastern United States (FL, NC, SC states; **Table 1**; **Figures 1A, D**). The last genetic cluster consisted of populations sampled from the Appalachian mountains (represented by southern Appalachian populations in western NC). This designation of collection sites into genetic clusters was one method to test whether or not the grouping of samples based on genetic identities was predictive of samples sorting into similar clusters when performing individual-based ordinations of microbial beta diversity (in addition to evaluating if microbial diversity differed among diseased vs. disease-free collection sites).

### Sequencing libraries and alignment to *E. pulchra* and *D. destructiva*

The GBS data were generated in studies by Pais et al. (2017); Pais et al. (2020). Some details are as follows. DNA extractions from collected leaf tissue were done using DNeasy Plant Mini kits (Qiagen, Inc., Valencia, CA, USA) and were prepared into three double-digest GBS libraries using PstI and MspI enzymes following the protocol of Peterson et al., 2012. Two reduced-genome libraries were sequenced on the Illumina 2000 Hi-seq platform in Pais et al. (2017) while the additional 137 collections were sequenced on the Nextseq platform in Pais et al. (2020). The 100bp paired-end (PE) sequences from Hiseq were sorted and trimmed of barcodes *in silico* using custom scripts described previously in Pais et al., 2017 and were further trimmed to be a standard length as the 64 bp paired-end Nextseq reads (also trimmed of barcodes). Additional filtering of low quality sequences (with more than five percent of bases’ quality below score of 20) was done prior to a sequence alignment procedure for separating putative fungal sequences from host plant sequences.

**Figure 2:**
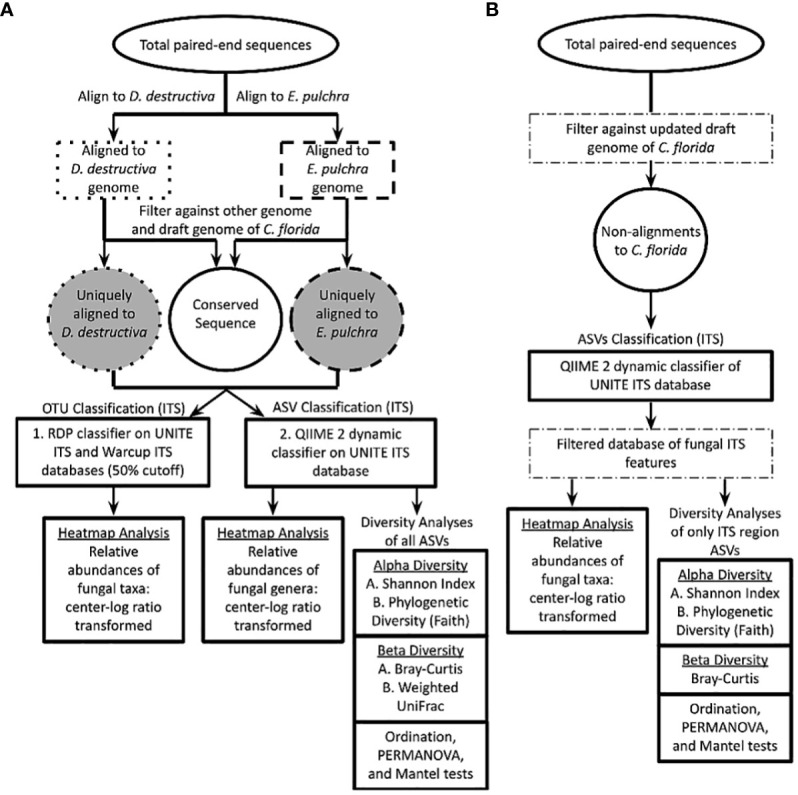
Workflow to determine proportion of total paired-end sequences aligning uniquely to genome of *Erysiphe pulchra* or *Discula destructiva.* Unique alignments to either fungal pathogen were applied to RDP Classifier and QIIME 2 platforms to obtain operational taxonomic units (OTUs) and amplicon sequence variants (ASVs) respectively. Relative abundance data of fungal taxa across sampling sites was each visualized using custom scripts for heatmap analyses on both RDP Classifier and QIIME 2 datasets independently. ASVs from QIIME 2 were applied for various diversity analyses diagramed. The UNITE ITS database diagrammed here was the primary source searched with RDP Classifier (Warcup ITS database also searched). QIIME 2 was searched against the UNITE ITS database as well. RDP classifier and QIIME 2 taxonomic search assignments against bacterial databases (e.g. Greengenes and RefSeq revealed less informative taxonomic assignments but are available in **Supporting Information** for reference. **(B)** An alternative set of analyses was done to address possible biases from the approach of part **(A)** This second approach analyzed only non-alignments to the updated *C*. *florida* genome and filtered results to only matches in the UNITE ITS database before performing further diversity analyses in QIIME 2.

The resulting fasta files were analyzed by Bowtie2 (Langmead and Salzberg, 2012) to extract fungal sequences, which were aligned uniquely to either available genomes of *E. pulchra* or *D. destructiva* initially (**Figure 2A**). The draft genome of *E. pulchra* was sequenced by Phillip Wadl (Wadl et al., 2019), and the draft genome for *D. destructiva* was sequenced and assembled by Ning Zhang, Guohong Cai, and their collaborators. Each draft genome was used for short read alignment to GBS paired-end sequences. Default parameters were specified to retain GBS sequences aligned to the genome of *E. pulchra* or *D. destructiva* for each sequence file corresponding to each sampled tree. The standard output report from Bowtie2 was exported to a file and modified into a table via custom R scripts to calculate the per-sample sum of paired-end sequences concordantly aligning with at least one hit to either genome. In order to reduce variation in results due to stochastic differences in sequencing effort among samples, each sum was then converted into a proportion based on total GBS reads sequenced per individual tree. Sequence reads aligning to both genomes of *E. pulchra* and *D. destructiva* or also aligning to an early draft genome of *C. florida* (Dogwood Genome Project, NSF ID: 1444567) were identified and parsed from results in subsequent analyses using Bowtie2. The proportion of total GBS sequences aligning concordantly and uniquely to one of the two pathogen genomes was quantified and recorded per individual tree before resultant PE, and mate sequences were applied to RDP Classifier for ITS-based taxonomic assignment (operational taxonomic units, OTUs) and to QIIME 2 for both ITS-based taxonomic assignment (amplicon sequence variants, ASVs) and diversity analyses.

### Isolating fungal sequences from non-alignments to *C. florida* host genome

In order to address possible biases arising from alignments to fungal genomes prior to QIIME 2 analyses, we reanalyzed data from non-alignments (GBS/RAD-seq reads that failed to align) to an updated version of the *C. florida* genome available in the Comparative Genomics (CoGE) Database and omitted aligning this alternative dataset with the fungal genomes previously mentioned (**Figure 2**). Processing, trimming, and alignment parameters were the same as previously mentioned for the reads aligned to the fungal genomes. For the sequence dataset obtained from non-alignments to the *C. florida* genome, we opted for implementing taxonomic assignments of ASVs and performing diversity analyses exclusively in QIIME 2 because the platform had become more widely accepted and adopted vs. RDP Classifier since our preliminary work. Downstream analyses of this second set of data (**Figure 2B**) were analyzed with similar parameters as the first dataset (**Figure 2A**) and reported subsequently—with exceptions detailed where merited.

### Taxonomic assignment and diversity analyses of fungal sequences

After consolidating single mate and PE fungal sequences (i.e. aligning uniquely to either *E. pulchra* or *D. destructiva* and not *C. florida* to reduce non-informative conserved sequences) for each tree sampled (**Figure 2A**), taxonomic assignments of fungal sequences were determined based on their most informative taxonomic rank. Taxonomic assignments were achieved with two popular platforms for studying microbiomes—RDP Classifier and QIIME 2. While both platforms were useful for identifying the relative abundances and putative identities of fungal sequences (based on OTUs with RDP Classifier vs. ASVs with QIIME 2), diversity analyses of fungal sequences were primarily and independently done with the QIIME 2 platform. Moreover, with our alternative set of analyses based on non-alignments to the *C. florida* genome (**Figure 2B**), we used exclusively QIIME 2—first to assign taxonomies to matches to the ITS region and then to use that filtered dataset to perform diversity analyses for comparison.

During the time of initial exploratory analyses using reads aligned to the two fungal genomes (**Figure 2A**, part 1), RDP Classifier was a common tool in metagenomics using naive Bayesian assignment to predict the identity of primarily ribosomal sequences to taxonomic units ranging from the level of domain to species (Wang et al., 2007). The RDP Classifier was run with the 2014 training version of the UNITE fungal internal transcribed spacer (ITS) database (Abarenkov et al., 2010; Kõljalg et al., 2013). This database frequently underwent expert curation and was a well-established source for taxonomic assignment of next generation sequence-derived OTUs, or operational taxonomic units (https://rdp.cme.msu.edu/download/posters/fungalITSreport_062014.pdf). While less informative in regards to identifying powdery mildew to species, the Warcup ITS database (Deshpande et al., 2016) was also used with RDP Classifier in order to compare taxonomic assignments of OTUs to other records of fungal taxa in *C. florida* (Farr et al., 1989; Miller et al., 2016) and visualize relative abundance differences between taxa across sampling sites (**Figure 2A**). Since our GBS sequences were shorter than 250 bp, we followed standard conventions recommended by authors of the RDP Classifier based on findings from Claesson et al. (2009). Namely, we applied an assignment confidence cutoff of 50% so that results below this threshold were binned as unclassified at a higher taxonomic rank. After using RDP Classifier to assign taxonomic ranks to fungal sequence OTUs within each sampled tree, we compared fungal composition among sampling sites based on taxonomic levels of class, order, genus, and species.

For taxonomic assignments of ASVs with QIIME 2, we applied our two different sets of aligned-process data (reads aligned to the fungal genomes and reads designated as non-alignments to the *C. florida* genome) into the classifier tool trained on a recent and curated ITS Sequence Database with (first analyses used Abarenkov et al., 2021; second analyses used Abarenkov et al., 2022: UNITE QIIME release for Fungi, DOI: 10.15156/BIO/2483915). For the approach involving alignments to fungal genomes (**Figure 2A**), taxonomic assignment and relative abundance data was collected separate from microbial diversity analyses on all ASVs. In contrast, the second approach using non-alignments to *C. florida* (**Figure 2B**) not only obtained taxonomic assignment data, but subsequent estimation and characterization of microbial diversity was performed on the filtered subset of data that consisted of matches exclusively to the UNITE ITS database (instead of estimating microbial diversity from all ASVs).

### Microbial diversity analyses

As a renewed effort to gather additional taxonomic assignment from our original data and perform downstream analyses derived from estimates of microbial alpha-beta diversity, the initial sequence files were reanalyzed in 2022-2023 using the QIIME 2 platform (Bolyen et al., 2019). Fungal sequences and *C. florida* non-alignments were reanalyzed against more recent versions of the UNITE ITS database (Abarenkov et al., 2021; Abarenkov et al., 2022)—this time using the classifier tool hosted by QIIME 2. ASVs were searched against a stringent and curated set of UNITE ITS hits derived from 97% and 99% clustering thresholds. Taxonomic classification in QIIME 2 using these stringent thresholds was expected to be more limiting compared to the initial 2016 search against the UNITE database using RDP Classifier (Eldred et al., 2021), but the primary purpose of using QIIME 2 was to more accurately estimate alpha and beta diversities of each sample and determine if fungal diversity of samples was related to location and disease conditions of collection sites. Findings from both RDP Classifier and QIIME 2 were visualized by modifying Python scripts (packages Pandas and Seaborn) to display relative abundance data (centered log-ratio transformed) onto heatmaps. These visualizations were done independently for each set of taxonomic assignments done with RDP Classifier (on UNITE and Warcup ITS databases) and QIIME 2 (on UNITE ITS database).

Using QIIME 2, we calculated multiple alpha and beta diversity measures for our samples. For our dataset of alignments to the two fungal genomes, we focused primarily on rarefied Shannon indices and Faith phylogenetic diversity indices for estimating alpha diversity and weighted UniFrac and Bray-Curtis distance matrices for representing beta diversity. The weighted Unifrac matrix incorporated phylogenetic distances between microbes when calculating dissimilarity measures whereas the Bray-Curtis matrix did not. For our QIIME 2 analysis of non-alignments to the *C. florida* genome (filtered to include only sequence matches to the UNITE ITS database), we estimated similar diversity indices and distance matrices as the former analysis procedure on the dataset of fungal genome alignments. However, due to artifacts of estimating phylogenetic diversity from a limited set of incomplete sequences of ITS data, we focused primarily on comparing beta diversity derived from a Bray-Curtis distance matrix. For estimating alpha diversity (Shannon and Faith indices) and beta diversity (Bray-Curtis and Weighted Unifrac distance matrices) from alignments to the fungal genomes, we specified a rarefaction/sampling depth of 1,341 features since this was the minimum threshold to maintain sample sizes of at least four per collection site. To achieve the same balance between useable reads and sample size per collection site, the minimum threshold set for the dataset consisting of ITS reads (from *C. florida* non-alignments) was permitted to be set higher (33,199 features) when estimating Shannon index, Faith’s phylogenetic diversity, and calculations of distance matrices (including Bray-Curtis and weighted UniFrac matrices). For understanding beta diversity and its relationship to the location and disease conditions of each collection site, we used various ordination procedures available with the QIIME 2 distribution and checked for statistically significant separation using PERMANOVA tests.

We also assessed the correlation between genetic distance and difference in beta-diversity of fungi among samples using full and partial Mantel tests. The genetic distance data were obtained from Pais et al. (2020) and the beta-diversity of fungi detected in each sample was estimated in this study as described above. We assessed the relationships between differences in fungal beta diversity and geographic distances of plant populations available in Pais et al. (2020). In these analyses, we also had the environmental factors controlled for analytically so that the relationship revealed was truly from genetic or geographic distances, excluding the influences of local environments. For evaluating the relationship of beta diversity with genetic differentiation of collection sites (and other distance matrices related to environmental dissimilarities across samples and differences in the proportion of fungal genome alignments per sample), we performed full and partial Mantel tests (9,999 permutations), which controlled for geographic distances and bioclimatic variation among collection sites.

## Results

### PE sequences aligning to pathogen genomes

**Figure 3:**
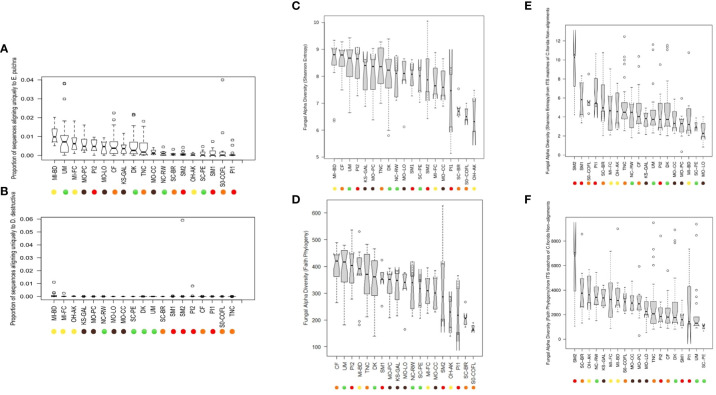
Notched boxplot represent differences in the proportion of GBS sequences uniquely aligned to either **(A)** the genome of *Erysiphe pulchra* (powdery mildew) or **(B)** *Discula destructiva* (dogwood anthracnose). Barplots of alpha diversity, including **(C)** Shannon Entropy and **(D)** Faith’s Phylogenetic Diversity by sampling site, are inferred from analysis of sequences on QIIME 2 and estimated from all ASVs obtained from alignment to two fungal pathogen genomes in addition to **(E)** Shannon Entropy estimates calculated only from ITS matches to non-alignments from *C*. *florida* genome and **(F)** Faith’s Phylogenetic Diversity estimates calculated only from ITS matches to non-alignments from *C*. *florida* genome. Boxplots depict minimum and maximum values (whiskers), outliers (dots), first quartile, median, and third quartile. The notches in each box correspond to the 95% confidence interval of each median value, and the width of each box corresponds to the square root of each collection site’s sample size. Sampling site IDs are colorized corresponding to ecoregions of sampling sites as visualized in **Figure 1**.

There were a total of 347,039,788 PE sequences from two libraries of Illumina Hiseq 2000 and one library of Illumina Nextseq that aligned to either of the two fungal genomes used. Each of the 312 samples had on average 1,112,307 PE sequences, albeit the standard deviation (SD) was high (SD: ± 827,105). Of the total GBS sequences, 1,572,066 concordant sequence pairs aligned uniquely to the genome of *E. pulchra* while 100,460 aligned uniquely to the genome of *D. destructiva* according to Bowtie2 reports. In other words, approximately 0.45% of total GBS PE sequences aligned uniquely to the genome of *E. pulchra* while 0.03% aligned uniquely to the genome of *D. destructiva*. However, sample-specific proportions of fungal to total sequences from each leaf extraction ranged as high as one sample having six percent of its sequences aligned exclusively to the genome of *D. destructiva*. In **Figures 3A, B**, the distribution of sample-specific proportions of fungal vs. total GBS sequences is reported with quartile and median values visualized for each sampling site. Lastly, when each component of an aligned PE sequence read was separated into individual mate sequences and pooled with lone mate alignments, there were a total of 3,493,202 pathogen-aligned sequences available for taxonomic assignment and diversity analyses using the RDP Classifier and QIIME 2 platform.

### PE sequences extracted from non-alignments to *C. florida* genome

Among the 312 samples used for comparisons with our two different alignment procedures, there were a total of 37,736,960 sequences that did not align to the updated *C. florida* genome and were imported into QIIME 2 after quality filtering. The average number of sequences per sample was 120,951 with a SD of ± 176,611, and after filtering this set with a classifier trained on the UNITE ITS database, there were a total of 32,739,391 sequences (average=104,934; SD=153,840; see **Supporting Information**) that were classified to fungi (or a more specific fungal taxa) and available for diversity analyses (described following our reporting of taxonomic assignments).

### Taxonomic assignments of sequences aligning to pathogen genomes

**Figure 4:**
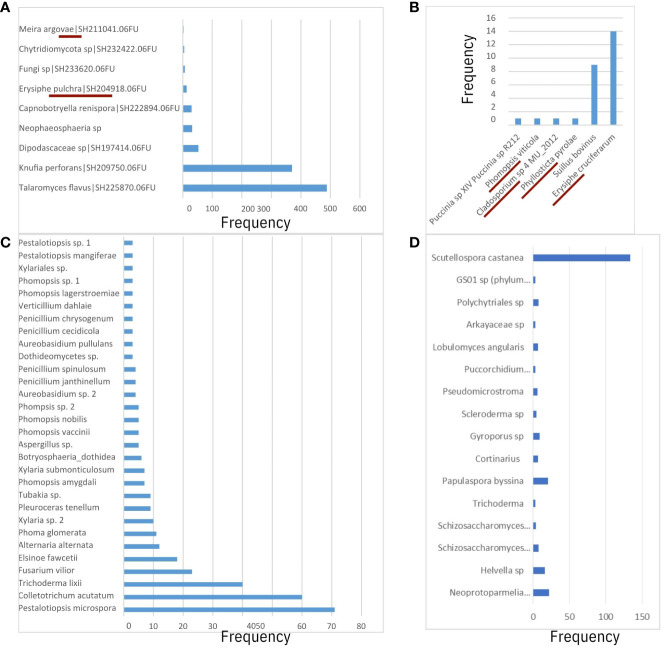
Comparison of fungal species-level operation taxonomic units (OTUs) between **(A)** 64-128bp GBS sequence matches to UNITE ITS database and RDP Classifier, **(B)** 64-128bp GBS sequence matches to Warcup ITS database and RDP Classifier, **(C)** Megablast results from Real-time PCR of ITS region from Miller et al., 2016. 64-128bp GBS sequence matches to UNITE ITS database using QIIME 2 did not reveal informative species-level predictions of taxonomy. Taxonomic ranks from this study **(A, B)** underlined in crimson if presence of fungi taxa confirmed in literature. **(D)** Genus-species ITS predictions from non-alignments to *C*. *florida* genome (as opposed to **(A, B)**, which were obtained from alignments to two fungal genomes).

**Figure 5:**
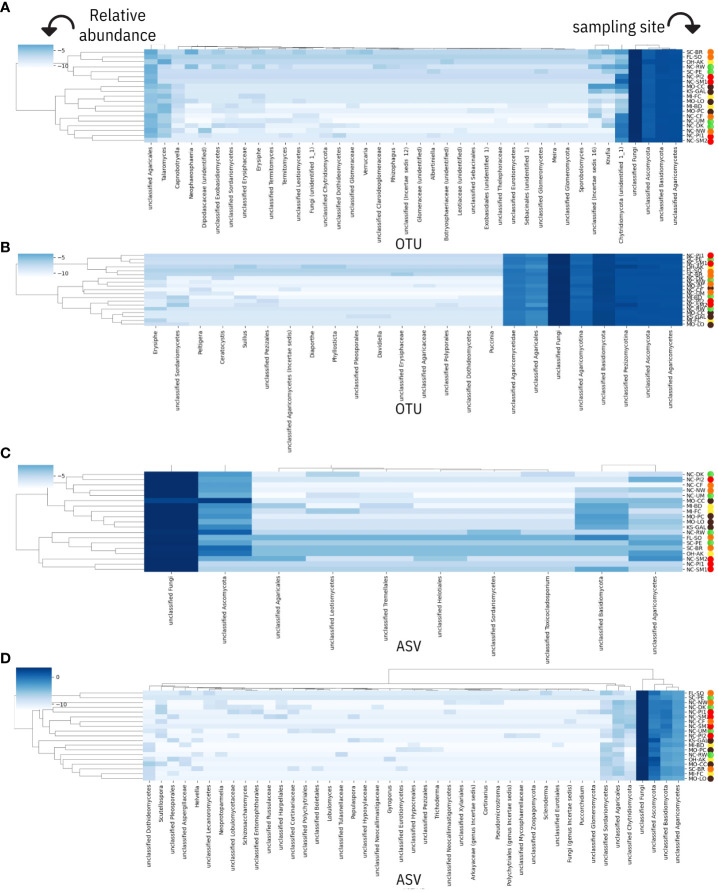
Genus-level fungal taxonomic assignments and their relative abundances within each *Cornus florida* sampling site. Each site is prefixed by its US state of origin and followed by a unique identifier as reported in **Table 1**. Sampling site IDs are colorized corresponding to ecoregions of sampling sites visualized in **Figure 1**. Results for heatmaps have been transformed (centered log-ratio) and are derived using the following: **(A)** RDP Classifier of UNITE ITS database, **(B)** RDP classifier of Warcup ITS database, **(C)** QIIME 2 dynamic classifier of UNITE ITS database (using sequences aligned to two fungal pathogens), and **(D)** QIIME 2 dynamic classification of non-alignments to *C*. *florida*. Taxonomic assignments by RDP Classifier are reported in operational taxonomic units (OTUs) while taxonomic assignments by the dynamic classifier used with QIIME 2 are reported as amplicon sequence variants (ASVs).

After running the RDP Classifier tool to assign taxonomic ranks to OTUs from pathogen-aligned sequences (one fastq file per sample), there were a total of 3,389,856 or 3,402,511 assignment counts to the domain of fungi using the UNITE ITS or Warcup ITS databases as training sets, respectively. Among classification results, the majority of assignment counts were unclassifiable (50%) while approximately 30% and 20% of results matched to phyla Basidiomycota and Ascomycota, respectively. For Basidiomycota, assignment counts belonged primarily to unclassified Agaricomycetes (**Figures 4**, **5**), and there was small evidence for the presence of basidiomycetous yeast-like fungi and rusts (e.g. *Meira* and *Puccini* sp.; **Figures 4A, B**). For the Ascomycota, a small proportion of taxonomic assignments (67 and 87 assignment counts for UNITE and Warcup ITS, respectively) indicated powdery mildew and dogwood anthracnose pathogen groups.

For the sequences obtained from alignments to the two fungal genomes and loaded in the UNITE ITS database (3,402,702), over 97% of the taxonomic assignments (reported as ASVs) were unclassifiable when searching against the UNITE ITS database with QIIME 2. Nonetheless, these sequences were retained for diversity analyses since our filtering procedure (**Figure 2A**) was able to functionally remove all sequences aligning to the plant genome of *C. florida* (possibly conserved sequences assigned as Viridiplantae that remained after filtering were approximately a third of a percent per sample) and there was no observable bias in the distribution of sequences not assigned to fungi across samples. For QIIME 2 (UNITE ITS) based results of the fungal genome alignments, approximately 2% of the sequence variants were assigned to the kingdom of Fungi. The fungal sequences that could be assigned to a phylum (approximately half a percent) represented Ascomycota almost exclusively by a factor of 37:1 relative to Basidiomycota (**Supporting Information**). When examining relative abundances of taxonomic assignments at the level of class using sequences aligned to the two fungal genomes and imported into QIIME 2, the following taxa of fungi were most prevalent in samples (from greatest to least at percentages less than a hundredth of a percent: Agaricomycetes, Sordariomycetes, Leotiomycetes, Tremellomycetes, and Dothideomycetes (**Supporting Information**). Taxonomic assignments more specific than the level of class were not substantial using the dynamic classifier on the UNITE ITS database of QIIME 2. The subsequent reports of taxonomic assignments related to powdery mildew are derived primarily from interpretation of RDP Classifier results (see *Patterns of microbial diversity* for further reporting of microbial diversity measures based on QIIME 2 analysis of ASVs regardless of taxonomic assignment).

From results based on the RDP Classifier-derived OTUs of both the UNITE ITS database (**Figure 5A**) and Warcup ITS database (**Figure 5B**), powdery mildews were identified in at least one sample from each collection site in the following sampling sites: MI-BD, MI-FC, MO-LO, MO-PC, NC-CF, NC-DK, NC-NW, NC-RW, NC-UM, and SC-BR, including assignment counts matching to the class of Leotiomycetes (**Figure 5A**) or the order of Erisiphales (**Figure 5A**). Sampling sites NC-DK, NC-UM, MI-BD, MI-FC, NC-RW, MO-PC, NC-NW, MO-LO, and SC-BR had ITS matches to family Erysiphaceae (**Figures 5A, B**), and sampling sites NC-DK, NC-UM, MI-FC, NC-RW, MI-BD, MO-PC, NC-NW, MO-LO, and SC-BR had genus (*Erysiphe*) level matches (**Figures 5A, B**). Each of the following seven collection sites had one sample that contained fungal sequences with species assignments to *E. pulchra*: NC-RW, NC-DK, NC-UM, MI-BD, MI-FC, MO-LO, and MO-PC (**Figures 5A, B**). Additional information concerning the specific assignment counts derived from the Warcup ITS or UNITE ITS database (from which the heatmaps of sampling sites in **Figure 5** have been based on) may be found within the **Supporting Information** of the **Supplementary Materials** provided with this study.

**Figure 6:**
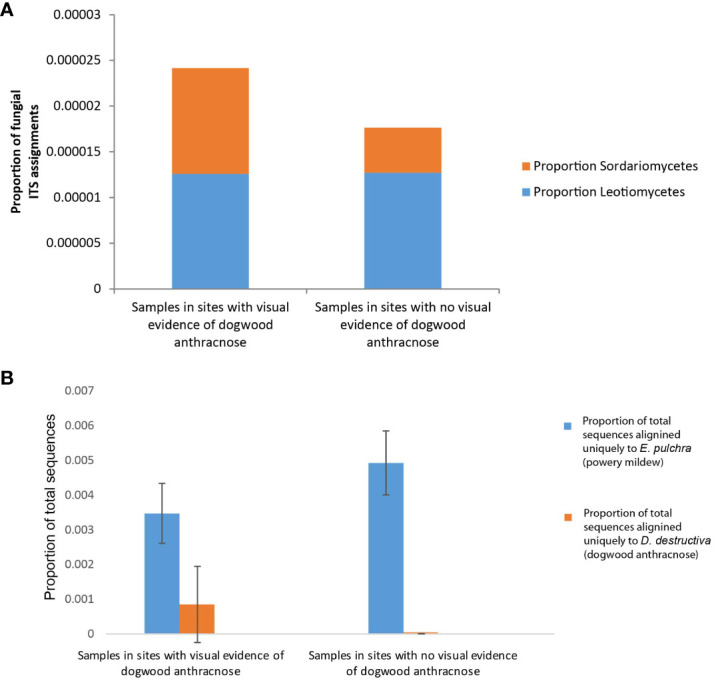
Bar graphs depicting phyllosphere fungal community composition differences between *Cornus florida* sampling sites with any visual evidence of anthracnose disease in early spring and summer (i.e. leaf blotting, necrosis, or branch dieback) and sites with no visual evidence of anthracnose disease. **(A)** We particularly note RDP Classifier results (specifically Warcup ITS results) of proportional differences in class-level operation taxonomic units (OTUs) for Leotiomycetes (blue) and Sordariomycetes (orange) since those taxa include powdery mildew and dogwood anthracnose, respectively. **(B)** In addition, see comparison of the mean proportion of total GBS sequences per sample aligned exclusively to either *Erysiphe pulchra* (powdery mildew; blue) or *Discula destructiva* (dogwood anthracnose; orange).

Fungal assignment counts that matched higher-level taxa inclusive of dogwood anthracnose were relatively small in proportion compared to fungal assignment counts associated with powdery mildew (see specifically Warcup ITS results, **Figure 6A**). Moreover, unlike *E. pulchra*, we did not find taxonomic assignments to the species of *D. destructiva* or the *Discula* genus within the OTU datasets generated by RDU Classifier (see Discussion under *Congruent findings of fungal community composition in C. florida* for interpretation of this absence). Taxonomic assignments reported as ASVs with QIIME 2 also did not recover any predictions of *D. destructiva*, its genus, or family, but taxonomic assignments to the inclusive class (Sordariomycetes) outnumbered the class inclusive of powdery mildew (Leotiomycetes) by six-fold. For RDP Classifier, we report the closest taxonomic assignments to other OTUs that include *D. destructiva* at the rank of class (Sordariomycetes), order (Diaporthales), and family (Diaporthaceae). Among assignment counts from both fungal ITS databases using RDP Classifier, sampling sites MI-BD, MO-LO, MO-PC, NC-CF, NC-DK, NC-NW, NC-PI2, NC-RW, NC-SM2, NC-UM, OH-AK, and SC-PE had trees containing fungal sequences assigned to Sordariomycetes, which included OTUs assigned to the plant pathogen family Diaporthaceae as well as OTUs assigned to two other genera in Sordariomycetes (*Ceratocystis* and *Albertiniella*; **Figures 5A, B**). Diaporthales and Diaporthaceae OTUs were attributed primarily to an assignment count to the genera *Diaporthe* (including its asexual classification *Phomopsis*; Gomes et al., 2013). This species was noted in sampling site OH-AK (**Figure 5**). Although datasets of fungal ITS assignments had large amounts of missing data, there were similar patterns between the proportions of Warcup ITS assignments to classes of Leotiomycetes and Sordariomycetes (**Figure 6A**) and the proportion of GBS sequences aligning uniquely to either the genome of *E. pulchra* or *D. destructiva* (**Figure 6B**).

### Taxonomic assignments from non-alignments to *C. florida* genome

Among the non-alignments (or sequences failing to align) to the *C. florida* genome that were searched against the UNITE ITS database using the classifier tool in QIIME 2, approximately 83% of assignments to phyla were Ascomycota with the majority of the remainder assigned as Basidiomycota. At the level of class, the majority of assignments that could be identified belonged to Agaricomycetes (approximately 97%), followed by Sordariomycetes (approximately 1.5%), Dothideomycetes (approximately 0.5%), and other trace amounts of other taxa (**Supporting Information**). We were not able to find taxonomic assignments of *Erysiphe* or *Discula* from the ITS matches obtained from the *C. florida* non-alignments. We did find at least 16 taxa at the genera and species level, that were broadly distributed among the higher-levels of taxa previously reported (**Figure 4D**). Combined with taxonomic assignments at higher taxonomic levels, there was some evidence observed for localized clustering of fungal composition (**Figure 5D**). Initial observations of clustering patterns by collection site were further characterized in diversity analyses reported subsequently.

### Patterns of microbial diversity for fungal genome alignments

**Figure 7:**
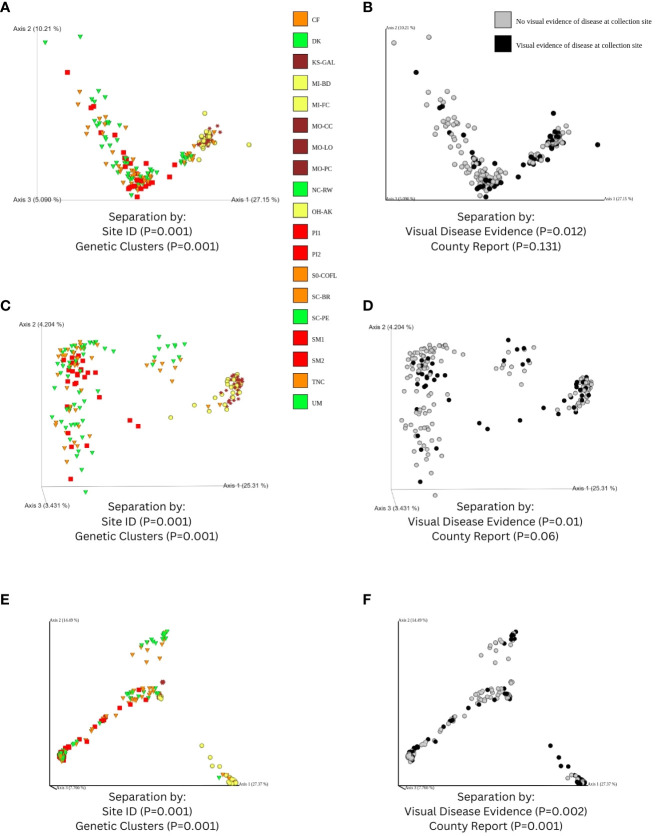
Ordinations of fungal diversity derived from sequences that were obtained from filtering-alignment to two fungal genomes, including: **(A)** visualization of a Weighted Unifrac distance matrix of samples colorized according to ecoregion (see **Figure 1**) along with shapes corresponding to genetic clusters identified from Pais et al., 2020; **(B)** an alternative coloring based on visual evidence of disease observed at collection site; and ordinations based on a Bray-Curtis matrix estimated from features of fungal genome alignments, colorized by **(C)** ecoregion and **(D)** visual disease category. Results of PERMANOVA tests overlaid on ordinations—validating that beta diversity across collection sites and genetic clusters was significant as well as based on classification of disease at collection site. Panels **(E, F)** parallel panels **(A, B)** respectively but represent results obtained from non-alignments to *C*. *florida* genome and filtering to include only matches to the UNITE ITS database. Moreover, **(E, F)** are visualizations of a Bray-Curtis distance matrix of ITS-based fungal diversity since phylogenetic-based matrices (i.e. Weighted Unifrac) presented artifacts of missing sequence data in the ITS region.

Data processed in QIIME 2 (regardless of taxonomic assignment) resulted in the calculation of rarefied alpha diversity averages by collection sites (**Figures 3C–F**) and generation of ordinations (**Figures 7A–F**) based on dissimilarity matrices of microbial composition (beta diversity), accompanied by statistical tests (e.g. PERMANOVA and full-partial Mantel) to evaluate the relationship between microbial composition within samples and the geography, disease conditions, and bioclimatic variables, and genetic differentiation experienced by sampled populations of the host plant species *C. florida*. While there were significant differences in rarefied Shannon indices and Faith’s phylogenetic diversity indices between collection sites, there wasn’t a clear pattern of differences based on ecoregion (**Figures 3C, D**), which corresponded with genetic clusters of *C. florida* (**Figures 1A, D**). However, analyses of beta diversity (more specifically weighted Unifrac) were more informative, showing clear separation of groups defined by collection site, genetic clustering of host, and visual evidence of disease at collection site (**Figures 7A, B**). Ordination based on the Bray-Curtis matrix was largely consistent (**Figures 7C, D**).

**Figure 8:**
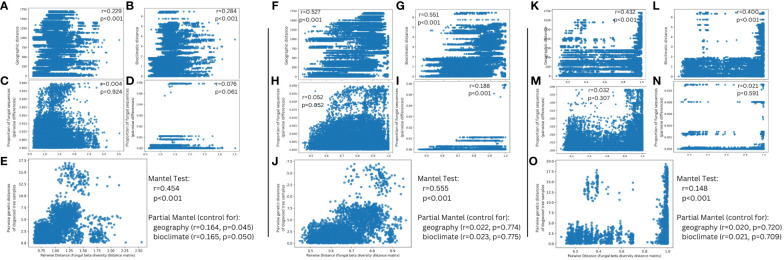
Mantel tests comparing correlation between fungal beta diversity and **(A, F)** a geographic distance matrix of *C. florida* trees sampled **(B, G)** Bioclimatic distances between samples corresponding to principal component 1 of **Figure 1A** **(C, H)** Pairwise distances of samples’ proportion of sequences aligning uniquely to *Erysiphe pulchra* **(D, I)** Pairwise distances of samples’ proportion of sequences aligning uniquely to *Discula destructiva*, and **(E, J)** Genetic differentiation of *C. florida* trees as estimated from Pais et al., 2020 after controlling for geographic and bioclimatic distance. Beta diversity represented from panels **(A–E)** was based on a weighted UniFrac distance matrix estimated from all ASVs of alignments to two fungal genomes while beta diversity represented from panels **(F–J)** was based on a Bray-Curtis distance matrix estimated from all ASVs of alignments to two fungal genomes. Panels **(K–O)** parallel panels **(F–J)** respectively, but represent sequences obtained from non-alignments to *C. florida* genome and filtering to include only matches to the UNITE ITS database. Moreover, **(F–J)** are derived from analysis of a Bray-Curtis distance matrix of ITS-based fungal diversity since phylogenetic-based matrices (i.e. Weighted Unifrac) presented artifacts of missing sequence data in the ITS region, which resulted in largely non-significant associations when correlated to other distance matrices. Pearson r and p-values overlaid on plots of each correlation.

For Mantel tests, pairwise distances of microbial diversity were significantly correlated (P < 0.001, Pearson r > 0.2) to distance matrices based on geographic distance and bioclimatic distance (**Figures 8A, B, F, G**). They were not correlated to distance matrices based on the proportion of either sequences aligning uniquely to the genomes of *D. destructiva* or *E. pulchra* (**Figures 8C, D**), except for one Mantel test based on a Bray-Curtis matrix (**Figure 8I**). When controlling for either geographic or bioclimatic distance, the weighted Unifrac distance matrix of microbial diversity was significantly correlated to pairwise genetic distances inferred from Pais et al., 2020 (P ¾ 0.05, Pearson r > 0.16; **Figure 8E**). A similar Mantel test based on a Bray-Curtis matrix also revealed significant correlation to pairwise genetic distances, albeit not when controlling geographic or bioclimatic distance (**Figure 8J**).

### Patterns of microbial diversity of ITS sequences filtered from *C. florida* non-alignments

Fungal diversity estimates derived from *C. florida* non-alignments revealed several patterns including significant differences among collection sites and significant associations with geographic, bioclimatic, and genetic differentiation of the plant host populations. Differences in rarefied alpha diversity among collection sites were significant across a number of comparisons (**Figures 3D, E**). However, clear differences in fungal diversity among different genetic clusters-ecoregions was more readily observed from comparisons of alpha diversity among defined genetic clusters (**Supporting Information**) and ordinations and coloring of samples by their defined genetic cluster ecoregion as seen in **Figure 7E**. The same ordinations also showed some signatures of sample sorting based on visual evidence of disease (**Figure 7F**). PERMANOVA tests supported differences in fungal beta diversity between collection sites, genetic clusters, and grouping based on diseased vs. healthy collection sites and county occurrence of disease (P-values from 0.001-0.002 for all tests).

Mantel tests based on a Bray-Curtis distance matrix of fungal diversity (as estimated from non-alignments to the *C. florida* genome that were filtered to only include matches to the UNITE ITS database) were highly correlated to geographic distance (**Figure 8K**) and bioclimatic distance matrices (**Figure 8L**) with Pearson coefficients above 0.400 and statistical significance below a P < 0.001 threshold. There were no significant correlations to distance matrices based on either the proportion of sequences aligning uniquely to either the genomes of either *E. pulchra* (**Figure 8M**) or *D. destructiva* (**Figure 8N**). The Bray-Curtis dissimilarity matrix of fungal diversity did show evidence of significant correlation to genetic distances of samples inferred from Pais et al., 2020 (P < 0.001, Pearson r = 0.148), but after controlling for either geographic or bioclimatic distance, there was not enough evidence to support the significance of this correlation (**Figure 8O**).

### *In silico* analysis of predicted restriction cut-sites in UNITE ITS database and GBS library

**Figure 9:**
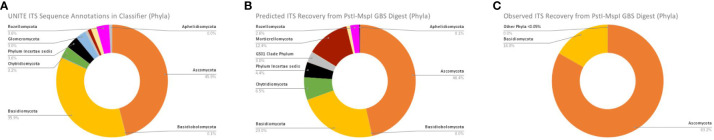
Comparisons of **(A)** Actual taxa annotated in UNITE ITS database; **(B)** Predicted ITS Recovery from an *in silico* PstI-MspI GBS digest of the UNITE ITS database; and **(C)** What was actually the observed ITS recovery from a PstI-MspI GBS digest and analysis of non-alignments to *C*. *florida* genome.

To ascertain biases in results stemming from aligning sequences to fungal or host plant genomes prior to metagenomics analyses, we report comparison of the fungal composition of our results against the reported annotated features in the UNITE ITS database in addition to reporting the predicted composition of features in the UNITE ITS database that would be recovered if performing an MspI and PstI double-digest according to our GBS protocol. For the clearest comparisons, we report differences in composition at the phylum level (**Figure 9**) with additional comparisons available in **Supporting Information**. Looking within the latest version of the UNITE QIIME release for Fungi used in this study, we found that approximately 46% of the annotated species hypotheses for the ITS region belonged to the phylum Ascomycota, followed by approximately 36% Basidiomycota, and trace amounts of other phyla at or below 3% each (**Figure 9A**). Predictions from our *in silico* digestion and size selection of the UNITE ITS database indicated that approximately 46% of the sequences recovered would be annotated as Ascomycota, followed by Basidiomycota (23%), Mortierellomycota (approximately 12%), Chytridiomycota (approximately 7%), and trace amounts of other phyla (**Figure 9B**). As previously reported, our observed results from our analysis of sequences that were first obtained by filtering sequence files against two fungal pathogen genomes (*E. pulcra* and *D. destructiva*) indicated that Ascomycota outnumbered Basidiomycota in features matching the UNITE ITS database by a ratio of 37:1. When performing analyses of sequences obtained only from non-alignments to the host plant (*C. florida*), the composition of observed matches to the UNITE ITS database (**Figure 9C**) showed approximately 83% of the matches belonged to Ascomycota with nearly 17% of the remainder belonging to Basidiomycota (other trace amounts of other Phyla below 0.05%).

## Discussion

The presence of fungal or microbial endophytes is often seen as a challenge to be controlled for (Schmieder and Edwards, 2011) when studying DNA from host systems such as plants (Laurin-Lemay et al., 2012), but an alternative paradigm warrants further consideration: “contaminant” sequences are helpful for describing fungal community composition across the range of host systems. Such a new approach would be helpful for further understanding the complex microbe-microbe interactions influencing plant hosts’ susceptibility to pathogens (Chaudhry et al., 2021). We have presented promising findings in support of adopting this new perspective. First, we determined that there was a sizable proportion of fungal sequences at least warranting filtering if study was to be concentrated on the host system. Our examination of microbial sequences within GBS libraries of *Cornus florida* reaffirmed the presence of several known phyllosphere fungal taxa associated with dogwoods and causal agents of powdery mildew in dogwoods (*Erysiphe pulchra*). There were approximately 1.5 million sequences aligned uniquely to the genome of powdery mildew (*Erysiphe pulchra*), and most notably, we discovered ITS sequences within our plants that matched OTUs of *E. pulchra* by genus and species (**Figures 4A**, **5A, B**). Moreover, we discovered that OTUs associated with *E. pulchra* were widespread across the range of *C. florida* (see **Figure 5B** of RDP Classifier/Warcup ITS results).

Our comparative analyses also revealed that host genetics of *C. florida* might be shaping patterns of microbial diversity based on correlation analyses accounting for effects of environment, disease, and location on microbial composition (**Figure 8E**). This result based on a Weighted Unifrac distance matrix of fungal genome alignments might be a byproduct of missing data in the alignment required to generate phylogenetic-based estimates of beta diversity. However, because of this concern we also incorporated diversity analyses on Bray-Curtis matrices (one for the fungal genome alignment dataset and another for the *C. florida* non-alignment dataset (**Figure 2**), which did not require phylogenetic based estimates possibly biased from missing ITS data. The full (but not partial) Mantel results applied to both Bray Curtis matrices did corroborate a connection between fungal beta diversity and genetic differentiation of the host (**Figures 8J, O**), and further work could be done in the future to determine if further consistent results might be revealed.

Although ITS-specific sequences were not detected to confirm the identity of *Discula destructiva* (the causal agent of dogwood anthracnose) due to lack of proper restriction sites, we did find patterns indicating that samples with visual signs of dogwood anthracnose disease had different microbial composition than healthier samples (**Figures 7B, D, F** and **Supporting Information**). This difference in fungal diversity between healthy vs. diseased samples may be related directly to the presence-absence of *D. destructiva* or may be correlated with environmental variables known to influence the distribution of dogwood anthracnose disease (Chellemi and Britton, 1992; Daughtrey et al., 1996; Hiers and Evans, 1997). The environmental variables could have directly influenced the significant differences in microbial diversity between healthy vs. diseased samples, which could have impacted the occurrence or absence of disease observed at collection sites and reported at the county level (**Figure 1A**). To some degree, we think that differences in the microbial community of the healthy plants led to different microbe-microbe and microbe-host plant interactions, which might have resulted in disease resistance or reduced disease susceptibility, a hypothesis that has been tested with a limited number of biocontrol agents in dogwoods (Osono and Mori, 2005; Osono, 2007; Mmbaga et al., 2008) and can be investigated further by experiments on phyllosphere fungal communities recently found in this study to be associated with *C. florida*.

Analysis of these microbial sequences in the leaves of dogwood samples, many of which belong to unculturable microbes, showed potential associations between environment, host genetics, and fungal community composition that can be explored further. Many of the diverse and ubiquitous microbes represented by such sequences likely play important roles in the host plant, possibly precluding particular plant pathogens as has been documented in other species (Arnold et al., 2003; Douanla-Meli et al., 2013). Through analyses of these “contaminate” sequences, we were able to gain insights into the relationships between genetic and geographic distances and differences in fungal communities in samples of *C. florida.* The finding of their significant positive relationships (**Figures 8A, B, E–G, I–O**) support important roles of both genetics or evolutionary divergence of host plants and geographic locations-environments on shaping fungal diversity. How endophytes map onto species-level genetic variation represents an understudied area (Harrison and Griffin, 2020). Our study in *C. florida* adds a new example to the scanty pool of literature on the topic (Liu et al., 2019; Zhou et al., 2023 and other studies from this issue), in supporting the role of genetic differentiation in shaping foliar fungal communities (see further discussion below).

### Congruent findings of fungal community composition in *C. florida*

The plant host interactions to fungi have been explored in a couple of other *Cornus* species (*C. sericea* and *C. controversa*) besides the flowering dogwood (Osono and Mori, 2005; Osono, 2007). The presence of fungal genera documented in these studies that interact with *Cornus* was also evidenced in our study, including Meira, Phomopsis, and Phyllosticta. The latter fungus was previously known as a cause of a relatively benign leaf spot disease in *C. florida* (Grand et al., 1975). Characterizations of fungal community composition within and on leaves of *C. florida* were previously conducted to document the following: pathogens, such as *Elsinoe cornii* (Jenkins and Bitancourt, 1948), *Septoria* (Farr, 1991), *E. pulchra* (Cook & Peck, Braun and Takamatsu 2000), and *D. destructiva* (Redlin, 1991); fungal competitors or mycoparasites (Mmbaga et al., 2008); and other fungal species (Farr et al., 1989; Miller et al., 2016). The most recent study of fungal diversity across the genus of *Cornus* by Zhou et al. (manuscript in this issue) revealed that *Erisyphe* constituted about one-third of ASVs among three samples of *C. florida*, and *Cladosporium* constituted about one-third of the ASVs of one sample. Our data also showed the occurrence of these fungal species in *C. florida* (**Figure 4** and **Figure 5**), albeit the relative abundances in this study were much smaller since the ITS region was not deliberately targeted for sequencing. Previous studies reported that a species from *Cladosporium* was abundant in the *Cornus* species studied and was considered a possible biocontrol agent for powdery mildew (Osono and Mori, 2005; Mmbaga et al., 2008). Although rare, we did detect the species but its relationship to powdery mildew was not confirmed based on our sequence data. The detection of *E. pulchra*’s ITS region and sequences of other regions of the species’ genome in leaf samples without apparent symptoms of powdery mildew was most surprising as it confirmed the presence of powdery mildew. This result demonstrates that our GBS data are not only suitable for studying *C. florida* but also useful to track the presence qualitatively of *E. pulchra*. Our data indicated that the occurrence of powdery mildew (i.e. *E. pulchra*) appeared widespread (**Figures 5A, B**) and was relatively unrestricted by the environmental conditions present in the sampled collection sites.

It is notable that ITS-based sequence assignments to *D. destructiva*, the pathogen causing dogwood anthracnose, the broader genus, and other known fungal taxa associated with *C. florida* (Miller et al., 2016; **Figure 4C**) were not detected in our study. However, this absence does not mean the species or other microbes known to inhabit *C. florida* is absent in our samples. The characterization of microbial diversity in this study is likely an underestimate. The absence of ITS sequences of *D. destructiva* in our data may be explained by one of the following four reasons: (1) Lacking the restriction sites for the enzymes of our choice for the GBS (i.e., MspI and PstI enzymes), referred to allele dropoff; restriction sites in the *D. destructiva*’s ITS region are necessary for the region to be sequenced; (2) the sequenced ITS region flanking the restriction sites are conserved in sequence among genera and thus, uninformative for resolving taxonomic assignment; (3) the ITS region was not represented in the draft genome of *D. destructiva*; (4) no *D. destructiva* was contained in DNAs extracted from the leaf samples in this study due to our efforts in selecting healthy leaves for genetic study of *C. florida* to avoid contaminated DNAs. We consider the first two reasons most likely while the last two reasons are unlikely because ITS regions are tandem repeats and abundant in the genome and many sequences were mapped uniquely to the genome of *D. destructiva*, suggesting the presence of the species. Although we were not purposefully targeting leaves with signs of anthracnose disease, we sampled leaves from several areas that were unmistakably affected by dogwood anthracnose. Our observation that diseased sites had only marginally higher relative proportions of Sordariomycetes-related sequences and sequences that aligned uniquely to *D. destructiva* (**Figure 6**) instead of *E. pulchra* and *C. florida* genomes suggests the trees from sites appearing to be healthy were likely not free of *D. destructiva* and may have been already infected by dogwood anthracnose. Although the differences in fungal composition amongst diseased vs. healthy tree sites were not conclusively attributed to dogwood anthracnose, our initial observation of patterns offered promising insights deserving further attention (**Figure 6B**). Here, we reiterate a cautionary disclaimer that some sequences aligning uniquely to *D. destructiva* may represent other members of Sordariomycetes, which may be worth further exploration via culture tests. However, since our measure is standardized across all individuals, our approach remains valuable for characterizing differences in relative abundance among populations of *C. florida*.

Another caveat to consider when analyzing sequences derived from non-alignments to *C. florida* (instead of aligning to the draft genomes of *E. pulchra* and *D. destructiva*) is that contaminants in the complete version of the *C. florida* genome might also have introduced dropout of taxa detected if those taxa were present as contaminants when the *C. florida* genome was being sequenced and assembled. This extra approach (**Figure 2B**) was in response to the concern of biasing downstream results from alignment to two fungal genomes, but as we see in **Figure 4D**, some of the taxa identified in our preliminary analysis (**Figure 2A**, **Figures 4A, B**) and in other studies of Cornus (Osono, 2007; Miller et al., 2016), were absent in our reanalysis. This underscores the importance of analyzing and comparing in different manners and how filtering and parameter choices may impact the conclusions of metagenomics studies. While the exact taxa identified from the two approaches did differ depending on the initial steps to align raw sequences, many of the trends were the same across our characterizations of beta diversity (**Figures 7A–D** vs. **7E–F**) and correlations to geographic, environmental, and genetic distances of the samples (**Figures 8A–J** vs. **8K–O**)—supporting the robustness of fungal diversity trends we observed.

### Influences on microbial diversity: environment vs. host genetics

One of the most interesting aims pursued by this study was to see if there would be a detectable signal of how the host plant’s genetic variation impacts the microbial composition inhabiting different populations of *C. florida*. There were collection sites that clearly had higher or lower alpha diversity vs. others (**Figure 3**) and true separation based on ordination of collection sites (**Figure 7**). However, correlations between different distance matrices were most informative in addressing what were the most important factors resulting in dissimilarity between collection sites in terms of their microbial composition. Geography and the associated weather-climate at collection sites were strongly associated with microbial composition (**Figures 8A, B, F, G, K, L**), underscoring the challenge of attributing any pattern to patterns of genetic differentiation of the host. Nonetheless, our study did find a connection between genetic differentiation of *C. florida* populations and the types of microbes inhabiting the differentiated populations (**Figure 8E**), suggesting there is clear variation that may be influencing (or be a consequence) of selective pressures characterized in prior studies of local adaptation in *C. florida* (Pais et al., 2017; Pais et al., 2020). Such a connection has long been explored but never, to our knowledge, using the partial mantel tests to control the effects of geographic distance and environmental variation (something we demonstrate in this study). As such, the framework provided by this study may show further utility for others interested in exploring the little known association between host genetics and diversity of symbionts within the host.

### Potential of GBS for metagenomics

The identification of important fungal pathogens and endophytes throughout an entire plant host’s range can be limited by the cost and effort of culture-dependent methods and the scarcity of expert taxonomists to identify inoculum in the field or lab from a broad array of fungi (Luecking, 2008). Fortunately, the ability to directly sequence and identify environmental or non-cultured samples—using metagenomics (Handelsman et al., 1998; Chen and Pachter, 2005)—has become more feasible with advancements in next generation sequencing (NGS) and growing databases for assigning taxonomic ranks to fungal OTUs using the ITS region (Schoch et al., 2012). In contrast to traditional Sanger sequencing, the use of barcoded multiplexing with restriction digest protocols such as GBS (Elshire et al., 2011) provides the ability to sequence many samples in one run on the Illumina platform, and the high-throughput volume of sequencing may allow for both sequencing of host individuals and the metagenomes they harbor. Data from such analyses provide more direct comparison between host genotypes and pathogen/microbial genotypes to study their interactions. While our initial study focused on host genetic diversity throughout the range of flowering dogwood (Pais et al., 2017; Pais et al., 2020), the data from GBS allowed us to conduct preliminary characterizations of fungal communities within *C. florida*.

However, the high stochasticity of sequencing the same loci in phyllosphere fungal communities from leaf tissue might lead to high proportions of missing data. Such missing data could bias phylogenetic-based estimates of fungal diversity, which underscores the importance of comparing phylogenetic-based diversity results with results from non-phylogenetic alpha and beta diversity estimates—an approach adopted in this study with largely similar results (e.g. see the following comparisons: **Figures 3C, E** vs. **3D, F**; **Figures 7A, B** vs. **7C, D**; and **Figures 8A–E** vs. **8F–J**. Beyond missing data due to stochasticity, only fungal taxa that had the appropriate GBS-associated restriction sites within the variable ITS1-2 regions (such as *E. pulchra*) were both detectable and identifiable to species. Fungal taxon and allele dropoff due to mutations at restriction sites of the ITS regions were expected. We checked the UNITE ITS database for the restriction sites of the two enzymes (MspI and PstI) used in our GBS libraries preparation and found that the proportion of taxa expected to contain the proper restriction sites (**Figure 9B**) did not vary considerably from proportions of taxa reported to comprise the UNITE ITS database (**Figure 9A**). When looking at the observed ITS recovery from taxonomic assignments to our actual samples, we found that analyzing the non-alignments to the *C. florida* achieved a ratio of Ascomycota to Basidiomycota (**Figure 9C**) closer to the expected ratio in the UNITE ITS database compared to the 37:1 ratio of Ascomycota to Basidiomycota estimated from analyzing sequences filtered from initially aligning GBS data to the genomes of *D. destructiva* and *E. pulchra*. While the latter procedure proved optimal for uncovering meaningful relative abundance data of *Erysiphe* (**Figures 4A, B**, **5A, B**), the former procedure (searching for ITS matches to the non-alignments of *C. florida*) likely introduced less bias for downstream diversity analyses, albeit many of the compared patterns were largely the same (e.g. see **Figures 7A–D** vs. **7E, F** and **Figures 8A–J** vs. **8K–O**). Therefore, the use of GBS of host plants to characterize microbial communities in host plants should be done carefully and account for these variables when interpreting the results. Nonetheless, we argue that microbial metagenomes can be explored by others with existing GBS datasets from other host systems. Moreover, if researchers wish to purposefully target symbionts and parasites living within their studied host systems, they can explore restriction site diversity and select restriction enzymes from GBS protocols that maximize throughput in sequence libraries of the host study system while targeting informative portions of associated metagenomes. In particular, we strongly recommend an *in silico* analysis of the ITS database be done when setting up the design of a GBS experiment to identify enzyme pairs that would give a more complete representation of the ITS segments useful for taxonomic analysis of fungal communities.

## Conclusion

Our analyses of fungal sequences in the GBS data of the flowering dogwood populations reaffirmed the presence of several known phyllosphere fungal taxa associated with dogwoods and an increasingly problematic pathogen [powdery mildew (*E. pulchra*)] in addition to revealing important drivers on microbial diversity among *C. florida* populations—particularly a significant effect that host genetic differentiation may have in shaping variation in the microbiome of samples. Even though we were not able to confirm the identity of the dogwood anthracnose pathogen to genus using our two different approaches, we did identify significant differences in fungal diversity between healthy and diseased samples, and differences in fungal diversity of samples also reinforced ordination patterns reflective of the differences between collection sites. Our comparison of alignment procedures (i.e. aligning to fungal genomes or host genome) prior to metagenomics analyses, did reveal differences in taxa identified. While the fungal genome alignment procedure showed better performance for tracking the relative abundance of known phyllosphere fungal taxa and pathogens of *C. florida*, the host genome-alignment procedure characterized overall patterns of fungal diversity that were largely the same as the fungal genomes alignment procedure. Moreover, the ratio of higher-level taxa identified from the non-alignment procedure was most similar to the proportion of taxa predicted to be recovered from digestion of the ITS region of many fungal species (represented in the UNITE ITS database) with the same restriction enzymes used for our preparation of GBS libraries. In totality, our results and comparisons from different approaches provide a number of caveats and suggested guidelines to take into consideration for other researchers designing or reexamining sequence experiments with the aim of exploring microbial patterns latent in their study system.

## Data availability statement

The original contributions presented in the study are included in the article/**Supplementary Material**, further inquiries can be directed to the corresponding author/s.

## Author contributions

AP: Conceptualization, Data curation, Formal analysis, Investigation, Methodology, Visualization, Writing – original draft, Writing – review & editing. JR: Supervision, Writing – review & editing. RW: Supervision, Writing – review & editing. QX: Supervision, Writing – review & editing, Funding acquisition, Resources, Project administration.

## Footnotes

1
Han, G., Wang, X., and Qiu, G. (2021). Mining endophytic microbiome information from plant and animal transcriptome data. *Bioinformatics*. doi: 10.1101/2021.05.07.443205
