# Supplementary figures and images for: Metagenomic study reveals hidden relationships among fungal diversity, variation of plant disease, and genetic distance in Cornus florida (Cornaceae)
Source: Front Plant Sci. 2024 Jan 11;14:1282188. doi: 10.3389/fpls.2023.1282188 (PMC10809005; doi:10.3389/fpls.2023.1282188)

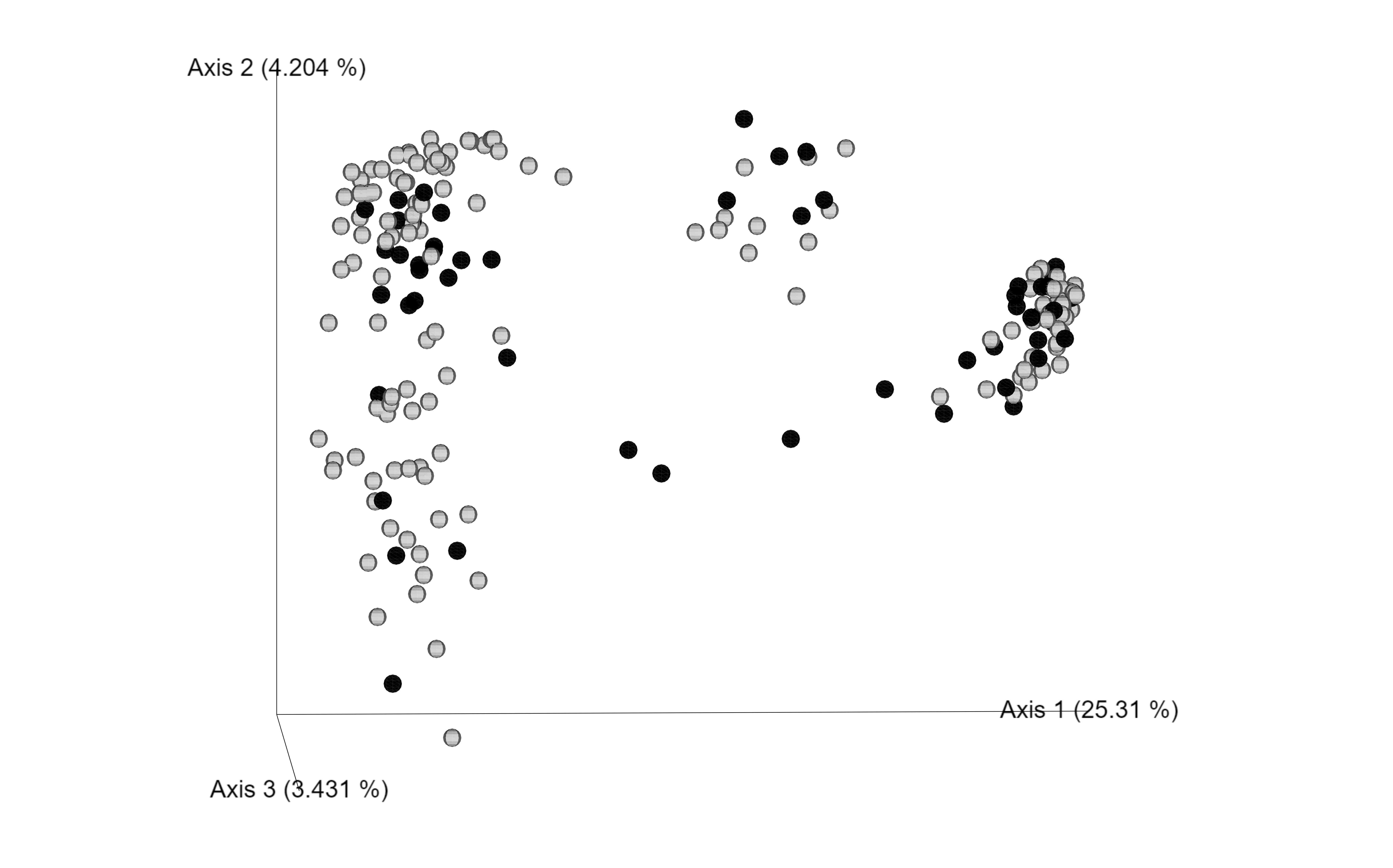

Supplement: Supplementary file 3 [file DataSheet_3.zip › Nov2023_Re-submit_RequestedAnalysis/New_outputs_to_unify/EPULDDESAlignedFeatures-BrayCurtis/Ordination-Permenova_Results/ToBeNewFig7C/emperor (4).png]

Fungal Alpha Diversity (Faith Phylogeny)

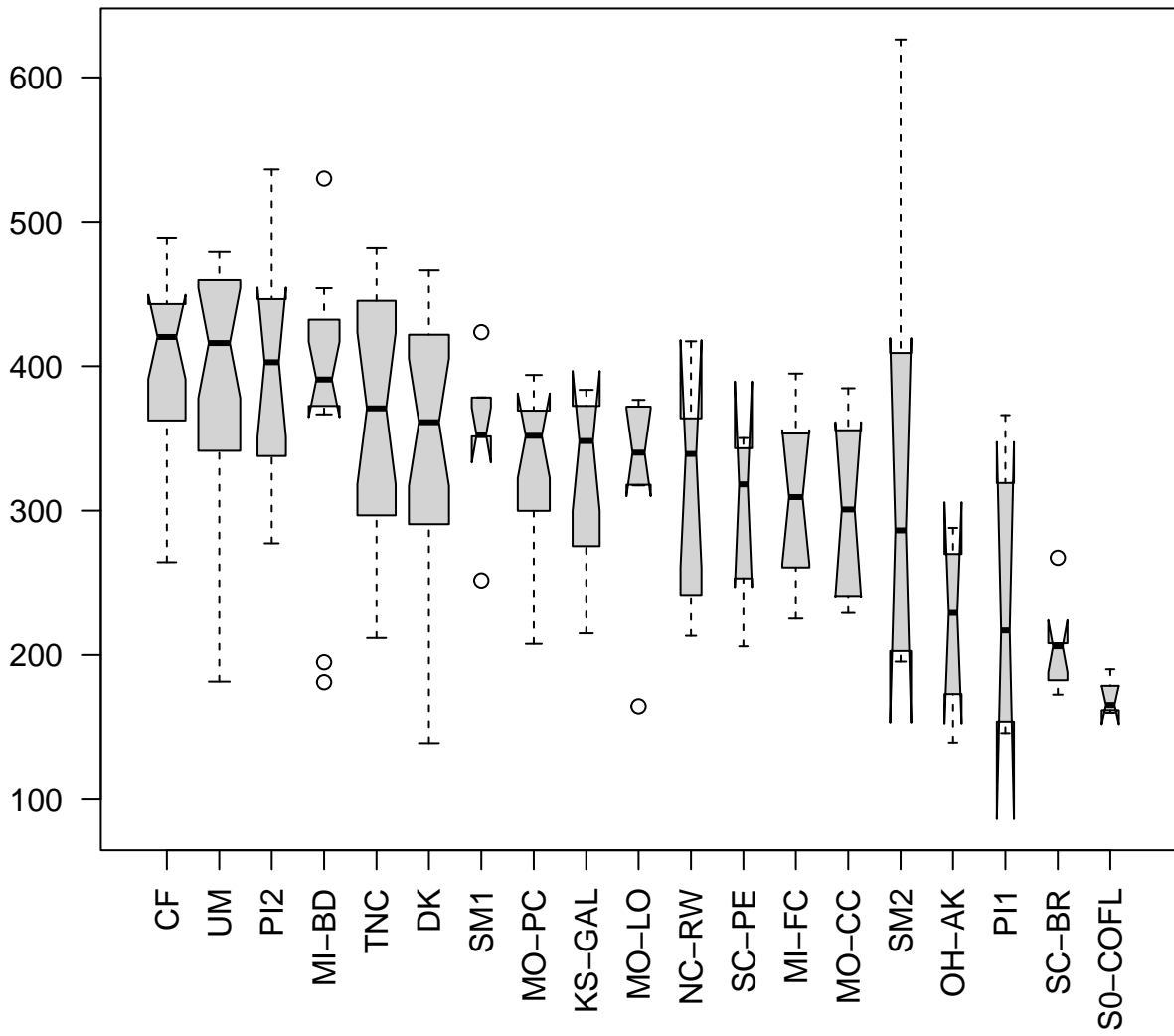

Supplement: Supplementary file 3 [file DataSheet_3.zip › Nov2023_Re-submit_RequestedAnalysis/New_outputs_to_unify/EPULDDESAlignedFeatures-WeightedUnifrac/Nov23RenalysisPDofEPULDDESAligns.pdf]

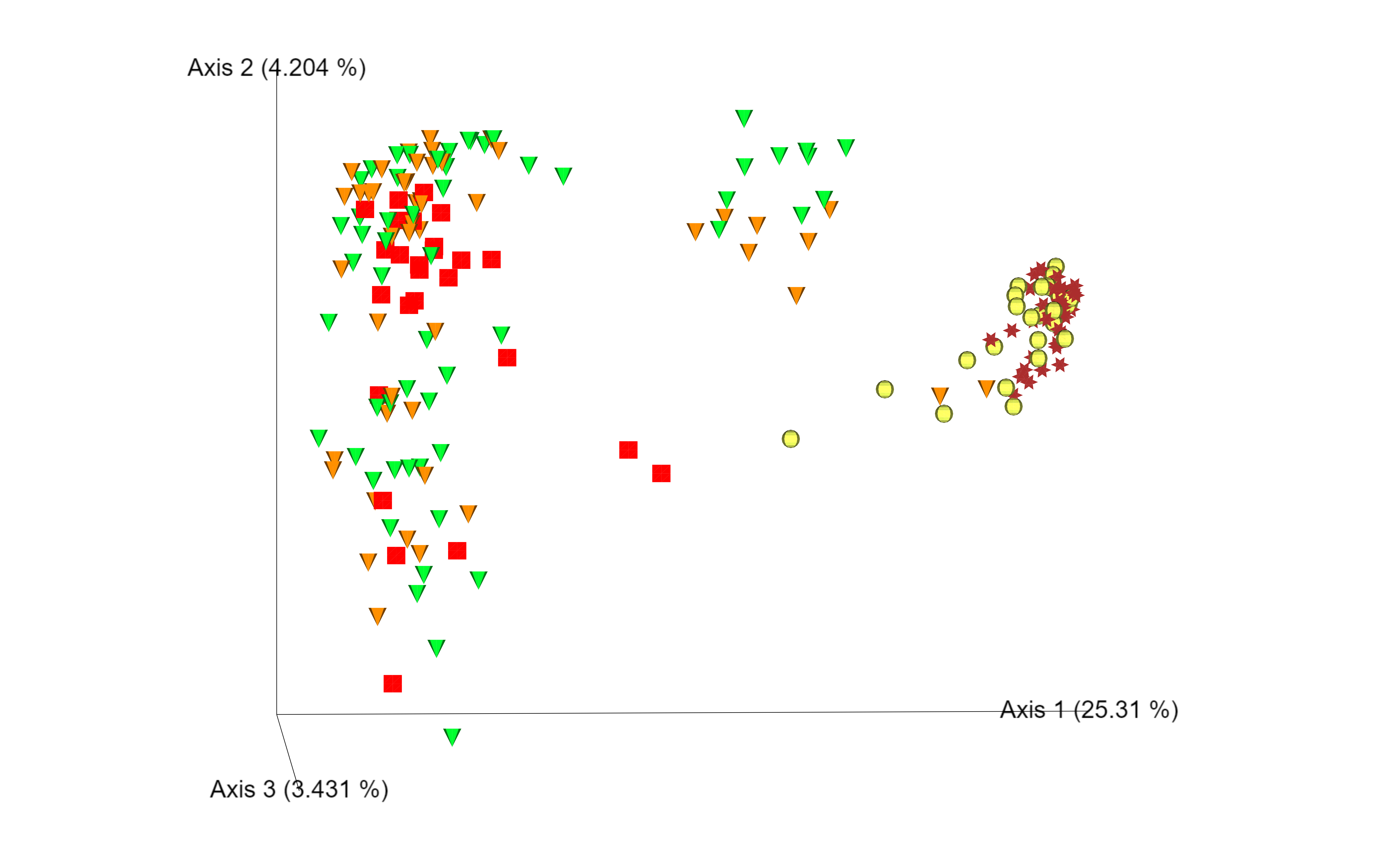

Supplement: Supplementary file 3 [file DataSheet_3.zip › Nov2023_Re-submit_RequestedAnalysis/New_outputs_to_unify/EPULDDESAlignedFeatures-BrayCurtis/Ordination-Permenova_Results/ToBeNewFig7B/emperor (3).png]
