# Supplementary material for: Metagenomic study reveals hidden relationships among fungal diversity, variation of plant disease, and genetic distance in Cornus florida (Cornaceae)
Source: Front Plant Sci. 2024 Jan 11;14:1282188. doi: 10.3389/fpls.2023.1282188 (PMC10809005; doi:10.3389/fpls.2023.1282188)
Supplement: Supplementary file 1 [file DataSheet_1.zip › Supporting Information - Approach 1 - Analyses of alignments to E.pulchra and D.destructiva/Preliminary QIIME2 Analyses.zip!/Preliminary QIIME2 Analyses/diversity_analyses-20230821T060028Z-001.zip!/diversity_analyses-20230821T060028Z-001/diversity_analyses/--p-sampling-depth 1341/Justification.docx]

This is the maximum sequence depth to maintain sample sizes of at least 4 per collection site and retain 265,518 (8.32% of 3,190,596) features in 198 (64.50% of 307) samples.

This is the closest setting we could obtain given the limitations of studying contaminant sequences while approaching recommended minimum sample depth parameter of 2,000 for single-end reads (Caporaso et al 2011).

Reference: Caporaso, J. G., Lauber, C. L., Walters, W. A., Berg-Lyons, D., Lozupone, C. A., Turnbaugh, P. J., ... & Knight, R. (2011). Global patterns of 16S rRNA diversity at a depth of millions of sequences per sample. *Proceedings of the national academy of sciences*, *108*(supplement_1), 4516-4522.
