# Supplementary material for: Metagenomic study reveals hidden relationships among fungal diversity, variation of plant disease, and genetic distance in Cornus florida (Cornaceae)
Source: Front Plant Sci. 2024 Jan 11;14:1282188. doi: 10.3389/fpls.2023.1282188 (PMC10809005; doi:10.3389/fpls.2023.1282188)
Supplement: Supplementary file 1 [file DataSheet_1.zip › Supporting Information - Approach 1 - Analyses of alignments to E.pulchra and D.destructiva/Preliminary QIIME2 Analyses.zip!/Preliminary QIIME2 Analyses/taxonomy_classification.zip!/taxonomy_classification/taxonomy_classification/UNITE_ITS/5-29-23_intermediate-output_files/1.classifier/citation for ]

Abarenkov, Kessy; Zirk, Allan; Piirmann, Timo; Pöhönen, Raivo; Ivanov, Filipp; Nilsson, R. Henrik; Kõljalg, Urmas (2021): UNITE QIIME release for Fungi 2. Version 10.05.2021. UNITE Community. <https://doi.org/10.15156/BIO/1264763>
